# Comparison of Tooth Widths, Arch Widths and Arch Lengths in Class-I Normal Dentition to Class-I and II Crowded Dentitions

**DOI:** 10.12669/pjms.37.2.3240

**Published:** 2021

**Authors:** Hafiza Zobia Shafique, Rumeesha Zaheer, Abdullah Jan, Ayesha Fazal

**Affiliations:** 1 Dr. Hafiza Zobia Shafique, BDS, FCPS-II Trainees, Orthodontics Department, Armed Forces Institute of Dentistry, Rawalpindi, Pakistan; 2 Dr. Rumeesha Zaheer, BDS, FCPS-II Trainees, Orthodontics Department, Armed Forces Institute of Dentistry, Rawalpindi, Pakistan; 3 Dr. Abdullah Jan, FCPS. Head of Department, Orthodontics Department, Armed Forces Institute of Dentistry, Rawalpindi, Pakistan; 4 Dr. Ayesha Fazal, MPH. Lecturer, Department of Community Dentistry, Margalla Institute of Health Sciences, Rawalpindi, Pakistan

**Keywords:** Angle Class-I malocclusion, Angle Class-II malocclusion, Dental crowding, Normal occlusion

## Abstract

**Background and Objective:**

Dental study casts play a vital role in the diagnosis and treatment planning of various orthodontic cases. This study was carried out to compare the tooth widths, arch widths, and arch lengths in Class-I normal dentition to those in Class-I and Class-II crowded dentition in an effort to improve treatment planning and to eventually reduce treatment duration.

**Methods:**

Total 170 patients, 12 to 40 years of age with a complete set of permanent teeth till 1^st^ molars; who presented to the Orthodontics Department at Armed Forces Institute of Dentistry (A.F.I.D), Rawalpindi from Sep 2019 to Feb 2020, were included in the study. Non-probability purposive method of sampling was used. The dental casts obtained were used to measure tooth widths, arch widths, and arch lengths. Subjects were classified into Class-I normal and Class-I and Class-II crowded occlusion and comparison of the sum of tooth widths, arch widths, and arch length discrepancies were determined among the three occlusion groups. Data was analyzed in SPSS version 21 and independent samples t-test was used to differentiate the variables of interest.

**Results:**

Out of 170 subjects, 73 (42.9%) subjects had Class-I normal occlusion while 97 (57%) had Class-I and Class-II crowded occlusions. No statistical difference was found between the occlusal groups with regard to the sum of tooth widths, inter-canine widths, inter-first premolar widths, inter-second premolar widths and inter-molar widths. However, a remarkable difference was observed between the occlusal groups with respect to arch perimeters and arch length discrepancies (p = 0.000 and 0.000 respectively).

**Conclusions:**

Results of the current study indicate that crowding of teeth occurs as a consequence of decreased arch perimeters which may lead to increased arch length discrepancies. However, no prominent difference was noticed in the sum of tooth widths and arch widths among different occlusal groups.

## INTRODUCTION

In the field of dentistry, dental records in the form of casts can play a vital role in critical analysis and treatment planning of any orthodontic case, by not only providing a 3-dimensional view of the dental occlusion but also allow precise measurements to be carried out.^1^,^2^ The measurements, that help in diagnosis and indicate the various treatment modalities,^3^ include the sum of tooth widths of both maxillary and mandibular dental arches and arch perimeters, which is the sum of dental arch lengths. Crowding and spacing in dental arches can also be calculated by measuring arch length discrepancies, which is obtained by subtracting the sum of tooth widths from total arch lengths (arch perimeters).^2^,^4^ Hence, the parameters of the dental arch that were considered in our study were arch widths, tooth widths, and dental arch lengths.

The stability of the outcomes of orthodontic treatment are also dependent on the dental arch form, of which the narrow-tapered arch form is the most prevalent.^5^ With an increase in age, every individual undergoes various remarkable changes in facial dimensions including the dental arch.^6^,^7^ These changes are more prevalent between the age group of 5 to 25 years, with the lower range, reflecting more changes in the females, while for males, more changes were reflected in the upper range.^8^ Albeit, these changes become mild over the period, but the process continues even in mid-adulthood on a small scale.^8^ Various studies have analyzed and proven these changes.^9^-^11^

An increased arch width was observed in males as compared to females when the two were compared in the mixed dentition stage. However, during the mixed dentition stage, major changes in arch width dimensions are attributed to environmental factors.^9^ Dental arches can be classified as Class-I, II, and III depending upon the arch size and unit length. According to this context, mandibular arch length in Class-II occlusion would be slightly shorter in length than in Class-I occlusion. In the same way, dental arches were smaller than normal for a crowded occlusal group when compared to the normal occlusal group,^11^,^12^ with Class-II div 1 occlusal group having the maximum degree of crowding among all other types of occlusion.

With the introduction of paperless work in the digital era, the urge to replace dental casts with computerized 3D imaging is emerging, in an effort to increase digitization of orthodontic records and to reduce dependence on physical dental casts. However, research has concluded that measurements on a dental cast with a digital caliper produce more accurate results than the 3D orthodontic models. Thus, we based our study on measurements recorded with a digital caliper by two observers, to reduce observer error.

Therefore, the objective of this study was to establish the relationship of tooth width, arch widths and arch lengths in Class-I normal, Class-I crowded and Class-II permanent dentition. The rationale of this study is to assess the relationship and to apply its use in finalizing the treatment plan and to subsequently reduce the treatment duration in orthodontic patients.

## METHODS

A comparative cross-sectional study was conducted over six months from September 2019 to February 2020 on approval of the Ethical Review Committee of Armed Forces Institute of Dentistry (A.F.I.D), Rawalpindi (905/Trg-ABP1k2). One-hundred and seventy patients, 12 to 40 years of age having a complete set of permanent teeth till 1^st^ molars; who presented to the Orthodontics Department, Armed Forces Institute of Dentistry (A.F.I.D), Rawalpindi, were included. Subjects with impacted/missing, supernumerary, deciduous, linguo-versed, bucco-versed, and transposed teeth were excluded. Subjects with developmental anomalies and facial asymmetry were also excluded from the study. Non-probability purposive sampling technique was used for the recruitment of samples and a sample size of 170 was calculated using the WHO calculator with the margin of error 5% and level of confidence 95%.

**Fig.1:**
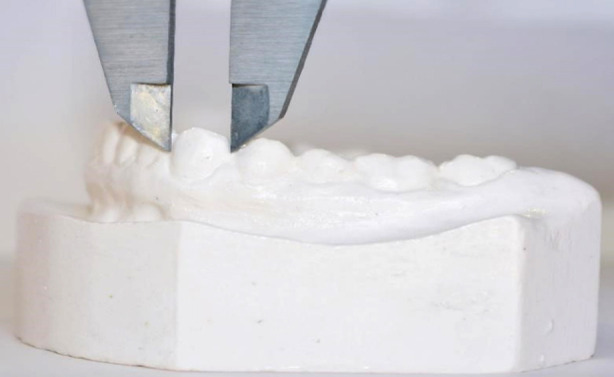
Mesiodistal dimension of teeth measured using Vernier caliper.

The materials that were used in the study were alginate, prefabricated impression trays, dental casts, and measuring instruments (Vernier caliper). Dental impressions were obtained using special impression trays loaded with alginate. Dental impressions were immediately poured in dental stone to get a replica of intraoral tissues and to make the dental casts. Industrial IP67 4Cr13 Stainless Steel Digital Vernier Caliper (WW-IP67), with the range of 0-150mm and resolution of 0.01mm, was used to measure tooth widths (Fig.1), arch widths and arch lengths on the dental casts. The tooth widths were determined by calculating the mesiodistal dimensions of each tooth at their contact points.

**Fig.2:**
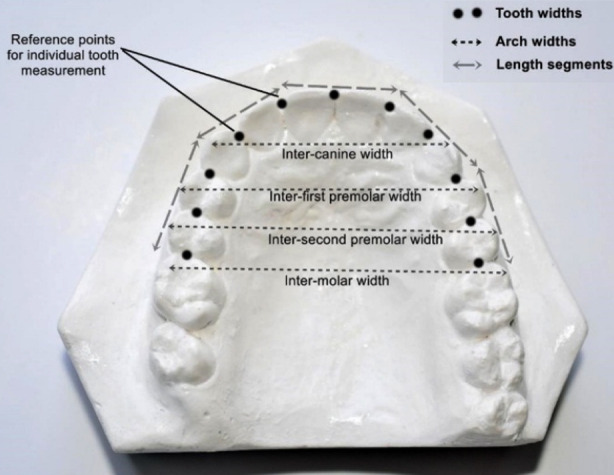
Reference points for measurement of tooth widths, arch widths (inter-canine, inter-first premolar, inter- second premolar, inter- first molar), and Arch length.

The arch length was divided into five segments from the mesial surface of 1^st^ molar of the right side around the arch to the mesial surface of 1^st^ molar of the left side. The points were recorded at the ideal location of the anatomic contact points of the teeth on the alveolar ridge (Fig.2). Arch widths were measured from the distance between cusp tips of canines, buccal cusp tips of premolars, and buccal cusp tip of molars as in Fig.2. To achieve accuracy and to reduce human error, two readings were taken by two different observers and then the average of the two was considered.

All of the casts were categorized into Class-I normal occlusion, Class-I crowded, and Class-II malocclusions. The mesiobuccal cusp of maxillary 1^st^ molar occludes on the mesiobuccal groove of the mandibular 1^st^ molar in normal Class-I occlusion group. The same relation was seen in Class-I crowded dentition but there is also crowding of more than 4mm. In Class-II dentition, the distobuccal cusp of the 1^st^ maxillary molar occludes at the mesiobuccal groove of the mandibular 1^st^ molars.

Data analysis was done using the software SPSS 21 (Statistical Package for Social Sciences, Chicago, USA). Independent samples t-test was used to differentiate the variables of interest (tooth mesiodistal widths, arch widths as well as arch lengths of the maxillary and mandibular arches). P-value was kept at 0.05.

### Ethical approval

Approval was received for this study from the ethical review committee of Armed Forces Institute of Dentistry, Rawalpindi (Reference number: 905/Trg-ABP1k2).

### Patients’ Consent

Informed consents were obtained from all patients.

## RESULTS

One-hundred and seventy subjects between the age group 12- 40 years (mean age 16.30±3.62) were included in the study. Out of 170 subjects, 73 (42.9%) were in Class-I normal occlusion, and 97 (57%) in Class-I and II crowded occlusions. Distribution of participants, on the basis of gender, was 65 males (38%) and 105 females (62%).

Mean, standard deviation and standard error mean of space required (sum of maxillary and mandibular tooth widths), arch widths (inter-canine, inter-premolars and inter-molar arch widths of both arches), maxillary and mandibular arch perimeters (total arch lengths), and arch length discrepancy, which is indicative of spacing or crowding in both the arches of Class-I normal, Class-I crowded and II crowded occlusal groups are illustrated in Table-I. The sum of tooth widths was found to lie within the same range with a slight difference, and arch widths were found to be lesser in crowded occlusal groups than in normal groups but the difference was neglible. However, a marked difference was observed in the arch perimeters of the two occlusal groups, with larger arch lengths for the normal occlusal group. Arch length discrepancies for both the groups were found to be within the range (less than ±4mm for the normal occlusal group and greater than -4mm for the crowded occlusal group).

**Table-I:** Mean, standard deviation and standard error means of the sum of maxillary and mandibular tooth widths, arch widths.

*Parameters*	*Class-I normal Occlusion N=73*		*Class-I and II crowded Occlusion N=95*		*p- value*

	*Mean ± SD*	*SE mean*	*Mean ± SD*	*SE mean*	
Sum of maxillary/ mandibular tooth widths	67.83±6.18	.51	67.60±7.53	.54	0.74
Maxillary/mandibular Inter-canine widths	31.73±4.63	.38	30.67±5.41	.38	.06
Maxillary/ mandibular Inter first premolar widths	38.93±4.87	.40	38.35±4.52	.32	.25
Maxillary/ mandibular Inter second premolar widths	43.74±5.37	.44	43.06±4.90	.35	.22
Maxillary/mandibular Inter-molar widths	50.47±5.66	.46	49.87±5.01	.36	.30
Maxillary/ mandibular arch perimeters	67.86±6.25	.51	61.83±10.88	.73	.000
Arch length discrepancies	.02±2.58	.21	-5.75±12.29	.88	.000

SD = Standard deviation

The levels of significance at 5% and the difference in confidence intervals among the variables of interest, i.e. the sum of tooth widths, arch widths, arch perimeters and arch length discrepancies among the maxillary and mandibular arches are indicated in Table-I. A statistically significant difference was shown between the two occlusal groups regarding arch perimeters and arch length discrepancies (p-value <0.05). However, no significant difference was found between the two groups with respect to the sum of maxillary and mandibular tooth widths, inter-canine, inter-first premolar, inter-second premolar, and inter-molar widths statistically.

## DISCUSSION

Correct diagnosis and prompt treatment are key to successful orthodontic treatment. In this study, the tooth widths, arch widths, and arch lengths have been assessed and compared between the two occlusal groups, Class-I normal and Class-I and II crowded occlusions. The null hypothesis made was that there is no difference between the two occlusal groups. There was statistically no significant difference found between the two occlusal groups in regard to sum of tooth widths (p-value: 0.74), inter-canine widths (p-value: 0.06), inter-first premolar widths (pvalue: 0.25), inter-second premolar widths (p-value: 0.22) and inter-molar widths (p-value: 0.30). However, there was a significant difference between the two occlusal groups with respect to their arch perimeters and arch length discrepancies (p-values: 0.000 and 0.000 respectively). So, we partially failed to accept the null hypothesis.

For a normal Class-I occlusion, the total sum of tooth widths needs to be in harmony with that of the arch lengths, so that minimum degree of arch length discrepancies exists within the two arches. The teeth in both the arches fall in their correct positions, leading to a balanced functional occlusion with minor and no discrepancies. In Class-I and Class-II crowded dentition, this discrepancy is more exaggerated, thus leading to an imbalanced occlusion. The sum of mesiodistal tooth widths, arch widths, and total arch lengths are considered to determine the crowding or spacing in both maxillary and mandibular dental arches. Through previous literature, tooth widths in both the arches were found to have significant effects on crowding, while arch lengths were found to have no association with the crowding.^2^ Moreover, in contrast to Class-I normal occlusion, the arch length discrepancies in crowded occlusion were more than 4mm, with decreased widths of both the dental arches.^13^ By these statements, we can surmise up that the cases may vary between different areas of studies depending upon the environmental and behavioral factors.

In this study, the arch perimeter, in both the dental arches, was found to be decreased in the crowded occlusal group (61.83mm±10.88) as compared to the normal occlusal group (67.86mm±6.25). However, the sum of maxillary and mandibular tooth widths was found to be slightly less for the crowded occlusal group (67.60mm±7.53) than for normal occlusal group (67.83mm±6.18). Likewise, arch widths had minimal to no difference between the two occlusal groups. In previous studies, the sum of tooth widths as well as arch widths was more in crowded occlusions than in normal Class-I occlusion, therefore an inverse relationship between tooth sizes and crowding had been established by previous studies. Nevertheless, no significant difference did exist between the tooth widths of crowded and non-crowded cases of dentition.^14^-^16^ Moreover, crown widths had no associated relation with the arch widths and arch perimeters.^17^ In the primary dentition, the tooth sizes are reduced as compared to the successors in the permanent dentition. If the arch perimeter and arch widths do not increase to compensate for this difference in the tooth sizes of both dentitions, crowding or spacing can occur. This is one of the leading causes of crowding or spacing in dental arches.^2^ If crowding exists in the mixed dentition, then crowding will most probably occur in the permanent dentition as well. Clinical examinations and history of the patients reveal that they had crowded occlusions in the mixed dentition stage as well.^17^ It has been concluded through the previous literature available, that extractions do not play a significant role in guiding the treatment plan for some of the parameters like inter-canine width and arch length discrepancies. In contrast to that, marked differences did exist for inter molar widths.^18^ The anterior Bolton’s analysis was insignificant in the previous studies when compared among the three occlusions.^19^,^20^ However, since the arch widths and arch lengths vary according to the literature available, careful evaluation for correct treatment and planning is needed.^21^

As shown above, we have concluded that analysis of arch lengths, arch widths, and mesiodistal widths of the teeth, help in the final treatment plan. The decision of whether extractions should be carried out to resolve the crowding of the arch is dependent on all of these determinants. No statistically significant difference was found in arch widths (inter-incisal and inter-canine widths) in previous literature reviews, while significant differences were observed in inter-molar widths in both extraction and non-extraction groups.^22^,^23^ The pre-treatment records of 202 patients were selected at random. Inclusion and exclusion criteria were applied, and the surviving records were divided into extraction (n =92) and non-extraction (n=110) groups. However, before orthodontic treatment, there is a significant difference in arch length discrepancy in both groups, with greater discrepancy seen in extraction cases that reduced significantly post-treatment.^23^ In the case of the arch perimeter, the previous literature review states that a reduced arch perimeter was seen in extraction groups in pre-treatment records. The decreased arch perimeter was compensated employing extraction of premolars, whereas, in non-extraction groups, an arch perimeter was not as reduced as in extraction groups.^23^

### Limitations of this study

Though attempts were made to minimize them, operator and instrumental errors couldn’t be completely eliminated.

## CONCLUSION

******The outcome of this study depicts that the arch perimeter greatly influences the position of teeth in the jaws. Decreased arch perimeters lead to increased arch length discrepancies resulting in crowding. However, no prominent difference was observed for the sum of tooth widths and arch widths between the two occlusal groups in our study.

### Authors’ Contribution:

**HZS:** Designed the research proposal and article writing.

**HZS & RZ:** Did data collection and data entry.

**RZ:** Reviewed and edited the manuscript. She is also responsible for the accountability or integrity of the study.

**AF:** Performed the statistical analysis and results interpretation.

**AJ:** Performed the final review and approval of the article..
